# The Nascent Iodine Treatment of Tuberculosis

**Published:** 1914-07-11

**Authors:** 


### MODERN METHODS SERIES.

### The Nascent Iodine Treatment of Tuberculosis.

The recently introduced method of treatment for tuberculosis, known as the "Intensive Nascent Iodine Treatment," has been tried by several clinicians; and the results which some of them have obtained are enthusiastically described in the *Practitioner.* It is obviously much too soon for any considered verdict upon the value and results of this method; but, inasmuch as it is now on trial, an acquaintance with its underlying principle is sure to be expected of the modern practitioner by some of his patients. Two reports of experimenters were published in the June number of the *Practitioner,* and the theory and practice of nascent iodine medication have been described in earlier issues of that magazine.

The hypothetical basis of the treatment is somewhat complicated, and a good deal of vague terminology and loose thinking is evident in the available accounts of it. Patients receive full doses of potassium iodide, so that their blood and tissues contain this substance in solution—that is, in a state of ionic dissociation. Then the patient takes chlorine water, containing potassium chloride to prevent its volatilisation. It is supposed that this chlorine will combine with the potassium ions, forming the perfectly harmless body potassium chloride, thus setting free nascent iodine in the tissues, where it is expected to have an antiseptic effect upon pathogenic microbes. It is believed that the chlorine is absorbed into the circulation as such. The hypothesis, it will be noticed, involves several assumptions of which any strict proof is not easy to supply; and there are further suppositions, advanced to explain the subsequent workings of the iodine, which are also accepted on very slender evidence. Thus, according to one of the writers in the *Practitioner,* nascent iodine has a stimulating effect upon the leucocytes, increasing their numbers in the blood and hastening their migration from the vessels into the tissues. This statement would necessarily imply that the gross number of the leucocytes is greatly increased, since both the blood and the tissues contain more of them; it also takes for granted that "nascent" iodine really is liberated in the tissues by this treatment. There is probably some confusion of thought here, and there is dogmatism of a much too positive kind in the same contribution. "Catalysis follows" (the liberation of iodine), we read, "any necrotic material first becoming liquefied, and then being either cast out or absorbed. . . . A vigorous reaction goes on, especially during the first week, attended by slight rise of temperature and some degree of iodism, which varies greatly in extent with the individual and with the amount of pathological tissue present. After the initial reaction has subsided, the nascent iodine continues to exert its powerful germicidal effects, and it is to these that the great value of this form of treatment in pulmonary tuberculosis is due."

This sort of pseudo-scientific verbosity carries exceedingly little weight, and it is a great pity that it should be indulged in. If the treatment is of real benefit, the theoretical explanation of it can wait awhile until the ground is rather firmer; if it is not, no amount of ingenious word-spinning will avail it in the long run. Happily there are several cases on record in which the practical upshot of the treatment appears to have been favourable, sufficient, in fact, to justify a further trial. Physical signs of pulmonary tuberculosis recede, it is stated, after a preliminary reactionary phase in which bronchitis is frequently experienced. The general condition, gauged by the aspect of the patient and by his own sensations, improves; this is often a fallacious test, as consumptives are habitually optimistic, and almost any novel treatment brisks them up for a time. Some gain in weight is described as occurring, but not uniformly. Cough, expectoration, and blood-spitting are also improved, but for the first fortnight may all be exacerbated. It would seem as if there must be some risk about a treatment which causes such a well-marked negative phase, but the possibility is pooh-poohed by the reporters. Night sweats are said to be made worse in some cases, another symptom which is explained away by an unconvincing form of words.

Tuberculous glands, again, are said to be very greatly benefited by the treatment. As an aid to diagnosis the method is also belauded, though it is not stated exactly what the aid is. If the phrase mean that an enlarged gland which disappears after "nascent iodine" treatment is thereby shown to be tuberculous, whereas one which does not is therefore non-tuberculous, an argument in a circle is being put forward. No other explanation of the diagnostic aid in question is obvious from the writer's language, though we hesitate to credit him with such a transparent and illogical absurdity.

Enough has been said to show that great caution is necessary in reference to the "intensive nascent iodine" treatment of tuberculosis. So far it has either been unfortunate in its apologists, or else it is fundamentally irrational. That fact need not deter anyone from a careful trial empirically, and there would seem to be a *prima facie* case established for some such trial. We cannot help thinking, however, that further trials, to carry weight with the profession, should be carried out by picked tuberculosis specialists with scientific reputations to lose, rather than by those who are at present so enthusiastic about the matter. We do not in any way reflect upon their energy, capacity, or trustworthiness; but there is a distinct risk that they may be allowing their hopes to run away with their judgment, and that the fight against tuberculosis may be hindered rather than helped. They have done service in directing attention to the possibilities of a new method, and should now be content to abide by the verdict of others.

